# Supplementary material for: Transmission of Methicillin-Resistant Staphylococcus spp. from Infected Dogs to the Home Environment and Owners
Source: Antibiotics (Basel). 2022 May 10;11(5):637. doi: 10.3390/antibiotics11050637 (PMC9137922; doi:10.3390/antibiotics11050637)
Supplement: Supplementary file 1 [file antibiotics-11-00637-s001.zip › Table S3 Metadata households.pdf]

Supplementary Table S3: Summary of metadata and medical history of the households A-H.

<table>
<tr><td>Household: A</td><td>Breed: English Bulldog</td><td>Sex: Neutered male</td><td>Age: 4</td><td>Diagnosis: Interdigital furunculosis, abscess</td></tr>
<tr><td colspan="5">DogA initially sought veterinary care for hematuria and swollen paws and was diagnosed with an enlarged prostate gland and deep interdigital pyoderma caused by MRSP 16 months prior to the initiation of this study. Dog A had a medical history of recurrent interdigital furunculosis and local dermatitis. Three bacterial swab samplings of the dog’s interdigital lesions, nostrils, and perianal region through the following year confirmed carrier status of MRSP. At the time of sampling for this study, the dog had interdigital furunculosis and a skin abscess on the neck. There were no other pets in the household, the dog had no contact with other animals, and it had not been outside of Scandinavia for the past 12 months. No antibiotics had been prescribed to the dog the past 12 months before the study. The dog was not allowed to be in the household’s beds or sofas. The dog was on a hypoallergenic diet. The owner of dog A worked within the health care service. None of the household members had been hospitalized, received antibiotic treatment, or been outside Scandinavia the past 12 months before the study.</td></tr>
<tr><td>Household: B</td><td>Breed: Hungarian Vizsla</td><td>Sex: Male</td><td>Age: 2</td><td>Diagnosis: Acute otitis externa</td></tr>
<tr><td colspan="5">Dog B sought veterinary care due to a case of acute otitis externa. Bacterial culturing confirmed MRSP and methicillin-susceptible Staphylococcus pseudintermedius (MSSP) present in the infected ear. The dog had been imported from Poland via Russia to Norway at six months of age. It had a medical history of recurrent seasonal deep pyoderma. It had previously been diagnosed with allergy to house dust mites (Dermatophagoides pteronyssinus and Dermatophgoides farina) and storage mites (Acarus siro and Tyrophagus putrescentiae). Before and during sampling, Dog B received subcutaneous Heska Immucept injections, and occasional topical interdigital treatments with hydrocortisone acetonate spray. The otitis externa was treated topically by cleansing the affected ear once a day with an ear cleanser and gel containing chlorhexidine. Dog B had not been outside of Scandinavia for the past 12 months. Neither had it been treated with antibiotics 12 months before the acute otitis externa nor during the period of samplings for this project. The dog was allowed to be in the household’s sofa but not in the beds. There were no other pets in the household, but the dog had sporadic contact with other dogs. It was on a hypoallergenic diet. None of the household members worked within the healthcare service had been hospitalized or been treated with antibiotics the past 12 months before sampling. The owners had been in Spain within the 12 months before the study.</td></tr>
<tr><td>Household: C</td><td>Breed: Chow Chow</td><td>Sex: Female</td><td>Age: 1</td><td>Diagnosis: Pyotraumatic dermatits</td></tr>
<tr><td colspan="5">Dog C was confirmed as an MRSP carrier after it had been in a long-term contact dog that suffered from multiple diffuse skin infections and urinary tract infections with MRSP, ESBL producing E. coli and Pseudomonas aeruginosa. The contact dog was euthanized approximately three months before Dog C was recruited to the study. Dog C was imported from Italy via Finland as a four-month-old puppy. It had a medical history of femoral head and neck ostectomy due to hip dysplasia at six months of age. In addition, it had been diagnosed with one case of acute otitis externa, interdigital furunculosis, and recurrent moist dermatitis localized to the tail. Dog C had an ongoing infection on the tail at the time of sampling for the study, and the bacterial culturing showed MRSP as the infective agent. The dog had received cefalexin and polymyxin B</td></tr>
</table>

antibiotics due to moist dermatitis and acute otitis externa within the past three months before it was recruited to the study. In addition, it had been treated locally with hydrocortisone and bathed twice a week with antifungal shampoo containing chlorhexidine and miconazole. The dog had recently begun on an allergy diet regime to rule out food allergy as the primary cause for the recurrent skin infections. A dog of mixed breed was in the household at the time of sampling. This dog normally lived in eastern Europe but was temporarily in the household. The contact dog had been screened for MRSP about a month before the sampling for this study and tested positive. This contact dog had no skin lesions or history of skin conditions. None of the dogs were allowed into the household's sofas or beds. None of the household members had been hospitalized within the past 12 months or worked within the healthcare service. One of the human household members had been treated with antibiotics within the past year but could not remember what kind. The owners had been to Germany, Poland, and Slovakia within the past year.				
Household: D	Breed: Staffordshire Bull Terrier	Sex: Male	Age: 1	Diagnosis: Surgical site infection
Dog D had two weeks before the initial sampling gone through a tibial-plateau-leveling osteotomy (TPLO) surgery. Symptoms of surgical site infection developed shortly after surgery, despite treatment with 400 mg cefalexin b.i.d post-surgically.<br>The dog was imported as a puppy from Poland and underwent surgery in the same country before returning to Norway, where he developed symptoms of infection. Bacterial culturing confirmed infection with MRSP as the infective agent. Dog D had not been treated with antibiotics nor received treatment for any skin or soft tissue conditions before this incident. It was allowed to stay in the households' beds and sofa. There were no other pets in the household. None of the owners worked within the healthcare service had been hospitalized or taken antibiotics the last 12 months prior to this study.				
Household: E	Breed: Rottweiler	Sex: Female	Age: 2	Diagnosis: Mastitis
Dog E sought veterinary care due to mastitis with onset about two weeks post-partum. Bacterial culturing confirmed MRSP as the infective agent. The litter consisted of 10 four-and-a-half-week-old puppies that showed no signs of infection. The dog had completed a two-week treatment with amoxicillin and trimethoprim and showed no signs of infection in the mammary gland at the time of the sampling for this study. The dog had not received antibiotics the past 12 months before this incident. The dog was allowed to be in the household's beds and sofas. It was regularly in contact with other dogs. None of the household members worked within the healthcare service, nor had been hospitalized or treated with antibiotics the past 12 months before this study. The owner had been in Turkey 5 months before the sampling.				
Household: F	Breed: Great Dane	Sex: Male	Age: 8 months	Diagnosis: Surgical site infection
Dog F underwent orthopedic surgery due to fractures in the radial and ulna bones in the left forelimb. It developed surgical site infection symptoms during the first days after surgery. The bacterial culturing was negative after the initial sampling, while resampling confirmed sparse growth of MRSP from the wound. The dog had been treated with cefalexin, amoxicillin, and ampicillin the week before it was diagnosed with MRSP. Enrofloxacin treatment was initiated after MRSP was confirmed. The dog had no previous history of dermatological issues. It had				

<table>
<tr><td colspan="5">received antibiotics five months before this incident due to a fractured toe and a subsequent friction wound from the bandage. However, the owner could not remember what kind of antibiotic. The dog had no contact with other animals outside of the household but lived together with another dog that suffered from seasonal superficial pyoderma. The contact dog had acute pyotraumatic dermatitis in the cheek region at the time of Dog F's incident. The dogs were allowed into the household's beds and sofas before the surgery. After surgery, Dog F's movement was confined to an enclosure in the living room, and the contact dog was not allowed inside the house during the convalescence period. Nor the dog nor the members of the household had been abroad the past 12 months before the surgical site infection. One of the household members worked within human health care, and one had been hospitalized within the past year. None of the owners had been treated with antibiotics for the past 12 months.</td></tr>
<tr><td>Household: G</td><td>Breed: Bullmastiff</td><td>Sex: Female</td><td>Age: 8</td><td>Diagnosis: Surgical site infection</td></tr>
<tr><td colspan="5">Dog G had undergone TPLO surgery due to a ruptured cranial crucial ligament four and a half years before joining the study. Six months after the surgery, it was diagnosed with purulent arthritis in the same knee. The bacterial culturing came back negative. The dog was treated with cefalexin for ten days and NSAIDS for 24 days and recovered from the infection. Approximately one year before it was recruited to this study, the owner noticed a wound on the right knee and sought veterinary care. The wound had healed at the time of the veterinary appointment, and the dog did not receive any treatment for the condition. A year later, the dog was diagnosed with a malignant mammae tumor without signs of metastasis and underwent a regional mastectomy. At the time of the surgery, the fistula on the knee was sampled, and the result came back positive for medium growth of MRSP. The dog developed symptoms of a surgical site infection and was then referred to the University Animal hospital for further medical examination. Bacterial culturing confirmed extensive growth of MRSP from the surgical wound and the knee fistula. The dog had no previous history of pyoderma, otitis externa or other skin related conditions before the surgical site infection. It was allowed into the household's beds and sofas. It had regular contact with other dogs outside of the household. None of the household members worked within the healthcare service, nor had been hospitalized, treated with antibiotics, or been outside of Scandinavia the past 12 months before joining the study.</td></tr>
<tr><td>Household: H</td><td>Breed: Rottweiler</td><td>Sex: Male</td><td>Age: 3</td><td>Diagnosis: Pyotraumatic dermatitis</td></tr>
<tr><td colspan="5">Dog H sought veterinary care due to a recurrent case of superficial pyoderma on the chin. It was diagnosed with pyotraumatic dermatitis two months earlier and was then treated with fusidic acid, amoxicillin, NSAIDs, and chlorhexidine. The condition worsened two months after the initial diagnosis, and culturing confirmed methicillin-susceptible S. pseudintermedius (MSSP), resistant to fusidic acid and amoxicillin, and methicillin-resistant Staphylococcus epidermidis (MRSE). The dog had a medical history with a complicated otitis externa the year before that recovered after treatment over six months consisting of ear cleansing with chlorhexidine, NSAIDs, and fusidic acid. The dog was allowed to be in the household's beds and sofas. It had no contact with animals outside of the household. It had recently been on a hypoallergenic diet to test for food allergies but was on a regular diet at the time of sampling. None of the household members worked within the healthcare service, had been hospitalized, had been treated with antibiotics or been outside of Scandinavia the past 12 months before sampling.</td></tr>
</table>
